# Mitochondria at Center of Exchanges between Cancer Cells and Cancer-Associated Fibroblasts during Tumor Progression

**DOI:** 10.3390/cancers12103017

**Published:** 2020-10-17

**Authors:** Lisa Nocquet, Philippe P. Juin, Frédérique Souazé

**Affiliations:** Inserm, CRCINA, Université de Nantes, F-44000 Nantes, France; lisa.nocquet@univ-nantes.fr (L.N.); philippe.juin@univ-nantes.fr (P.P.J.)

**Keywords:** cancer, cancer-associated fibroblast, mitochondria, metabolism, apoptosis, BCL-2 family proteins

## Abstract

**Simple Summary:**

Malignant cells and their supportive associated fibroblasts (CAFs) exchange various molecules that promote energy production, biosynthesis and therapy resistance by modulating mitochondrial activity and dynamics. We herein review molecular exchanges from CAFs to malignant cells that support tumor growth and therapy resistance, and we highlight the crucial role of CAFs mitochondria in this support. This implies (1) reciprocal mitochondrial control by malignant cells and (2) fibroblast activation. Finally, we discuss therapeutic strategies that could improve current therapies by targeting mitochondrial-mediated dialogue between the two cell types.

**Abstract:**

Resistance of solid cancer cells to chemotherapies and targeted therapies is not only due to the mutational status of cancer cells but also to the concurring of stromal cells of the tumor ecosystem, such as immune cells, vasculature and cancer-associated fibroblasts (CAFs). The reciprocal education of cancer cells and CAFs favors tumor growth, survival and invasion. Mitochondrial function control, including the regulation of mitochondrial metabolism, oxidative stress and apoptotic stress are crucial for these different tumor progression steps. In this review, we focus on how CAFs participate in cancer progression by modulating cancer cells metabolic functions and mitochondrial apoptosis. We emphasize that mitochondria from CAFs influence their activation status and pro-tumoral effects. We thus advocate that understanding mitochondria-mediated tumor–stroma interactions provides the possibility to consider cancer therapies that improve current treatments by targeting these interactions or mitochondria directly in tumor and/or stromal cells.

## 1. Introduction

Mitochondria have been implicated in tumoral progression since Otto Warburg described mitochondrial dysfunction associated with glycolytic activity increase even under normoxia as a tumor promoter in 1927 [1]. Since then, it has been shown that mitochondria, even impaired, still provide malignant cells with energy and biosynthetic precursors, and control redox homeostasis and resistance to apoptosis. Indeed, the intrinsic pathway of apoptosis relies on mitochondrial outer membrane permeabilization (MOMP) leading to caspases activation and subsequent loss of cell integrity. Thus, the mitochondrial apoptosis resistance process taking place up or downstream of MOMP is crucial to cancer cell survival.

Cancer cell interactions with others cell types, such as cancer-associated fibroblasts (CAFs), immune cells and endothelial cells, actively participate in tumor progression, including tumor growth, survival and invasion [2]. In particular, CAFs and tumor cells strongly dialogue via soluble factors, exosomes, extracellular matrix components and direct contacts [3]. The two cell types educate each other to adapt to their nutritional and signaling environment. Glycolytic CAFs have been shown to enhance the contribution of mitochondria to energy production and biogenesis in cancer cells, also promoting tumor progression. This process was called the “Reverse Warburg Effect” [4]. Here, we focus on both mitochondrial metabolic activity and the apoptosis resistance of cancer cells under CAFs control. Importantly, the metabolic dialogue between CAFs and cancer cells implies a reciprocal influence of cancer cells on CAFs metabolism, which participates in their pro-tumoral effects. Moreover, cancer cells have been shown to attract and activate fibroblasts via cytokines and growth factors [5]. Here we focus on the implication of mitochondrial regulation in fibroblasts activation signaling pathways. Importantly, we discuss the heterogeneity of mitochondrial activities within tumors and between tumors, highlighting the complexity of targeting the metabolic dialogue and mitochondria directly, by using drugs in combination with current treatments.

## 2. CAFs Sustain Cancer Cells Mitochondria

### 2.1. CAFs Reorganize Cancer Cells’ Mitochondrial Metabolism

Here, we focus on CAF/cancer cell metabolic interactions that impact malignant cells’ mitochondria.

CAFs have been shown to fuel cancer cells with organic and amino acids. Pyruvate is an organic acid at the crossroad between glycolysis and mitochondrial oxidative phosphorylation (OXPHOS). It fuels the tricarboxylic acid (TCA) cycle and subsequent mitochondrial respiration. CAFs can directly provide cancer cells with pyruvate (as shown in lymphoma [6]), and also indirectly by providing lactate (as shown in prostate cancer [7,8] and breast cancer [4,9]) or alanine (as shown in pancreatic cancer [10]), both latter metabolites being transformed into pyruvate via active lactate dehydrogenase and alanine aminotransferase, respectively. CAFs also fuel malignant cells with glutamine in glutamine-deprived conditions (as shown in ovarian cancer [11]), which is transformed into glutamate and then alpha-ketoglutarate to enter the TCA cycle and generate biosynthetic precursors. Of note, metabolites are not only exchanged from CAFs to cancer cells via their soluble forms since amino-acids and TCA cycle intermediates can be shuttled via exosomes, upregulating, in this case, glycolysis but reducing OXPHOS (as in prostate and pancreatic cancer cells [12]).

Thus, CAFs provide intermediate metabolites for malignant cells mitochondrial activity. More precisely, these metabolites fuel malignant cells’ TCA cycle, which feeds biosynthetic pathways to produce key precursors such as lipids, proteins and nucleic acids, thus promoting primary and metastatic cell growth [7,10,11]. In some of the studies, TCA cycle modulation induced by CAFs even leads to higher malignant cell oxygen consumption, reflecting mitochondrial respiration increase [8,10]. In addition, a CAFs-induced increase in TCA cycle activity is associated with primary patient malignant cell survival [6]. Of note, CAF-induced metabolite consumption is enabled by the concomitant upregulation of metabolic transporters, such as lactate transporter MCT1 (in prostate cancer cells [4,7,13]).

Beside fueling TCA, lactate promotes mitochondrial biogenesis. Indeed, lactate consumption by metastatic prostate cancer cells under CAFs-control, via shifting NAD+/NADH cell equilibrium toward NAD+ that is a substrate of Sirtuins (SIRTs) [14], activates SIRT1/PGC1α axis that promotes mitochondrial biogenesis and activity [8]. CAFs might also favor cancer stem cell traits as SIRT1 has been shown to regulate cell stemness [15], and as this phenotype mainly relies on oxidative phosphorylation (in ovarian and breast cancer [16,17]). Moreover, SIRT1/PGC1α axis is amplified by concomitant activation of proto-oncogene tyrosine-protein kinase Src due to TCA cycle deregulation-induced reactive oxygen species (ROS) production [8]. Interestingly, ROS production, which is elicited by both respiratory chain overload and mild respiration dysfunction, has been shown to induce Src activation, promoting tumor cell migration [18]. Mitochondrial ROS, related to CAF-induced metabolic reprogramming, could be involved in many other tumor progression mechanisms, since sustained ROS production promotes tumor proliferation, genetic instability and some treatments resistance [19].

Upregulated mitochondrial activity, associated with the downregulation of TCA cycle enzymes, can also lead to the accumulation of mitochondrial metabolites, called oncometabolites, when participating in tumor progression. For instance, CAF-induced mitochondrial fueling of prostate malignant cells leads to succinate and fumarate accumulation [8]. Importantly, succinate accumulation induces HIF1α stabilization and subsequent oncogenic epithelial–mesenchymal transition (EMT). Fumarate accumulation has also been shown to favor EMT of renal cancer cells via epigenetic modifications [20], thus promoting invasion.

**Figure 1:**
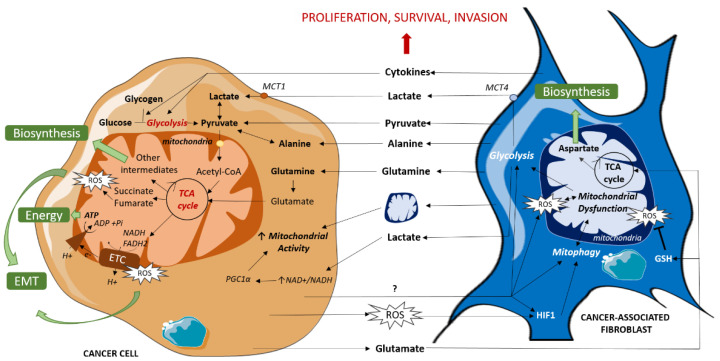
Metabolic dialogue between cancer cells and CAFs. CAFs fuel TCA cycle by directly providing cancer cells with organic (lactate, pyruvate) and amino acids (alanine, glutamine) or indirectly by enhancing glycolysis via cytokine release, resulting in an increase in mitochondrial activity which leads to energy and biosynthetic precursor production and redox state modulation. CAFs also enhance mitochondrial activity via mitochondrial transfers. In turn, cancer cells induce mitochondrial dysfunction in CAFs, mitophagy and ROS production, amplifying their mutual support. EMT: Epithelial Mesenchymal Transition, ETC: Electron Transport Chain, GSH: reduced glutathione, mtDNA: Mitochondrial DNA, MCT: MonoCarboxylate Transporter, ROS: Reactive Oxygen Species, TCA cycle: TriCarboxylic Acid cycle.

Some CAFs subpopulations have also been shown to reorganize cancer cell metabolism via secreting cytokines that favor glycolysis and TCA cycle intermediates production, resulting in tumor growth and invasion in breast and pancreatic cancers [21]. A similar cytokine-based interaction was shown in ovarian cancer, implying IL-6, CXCL-5 and CXCL-10, which promotes self-stored glycogen utilization by cancer cells to fuel glycolysis and subsequent mitochondrial activity [22]. CAFs’ metabolic impact on cancer cells is summarized in Figure 1.

### 2.2. CAFs Provide Intact Mitochondria to Support Cancer Cells Mitochondrial Activity

Mitochondrial transfers occur between cancer cells and different cell types and are thought to happen in many cancers to optimize or repair the malignant cells’ metabolic machinery [23], promoting cancer progression. Indeed, mtDNA transfer from in vivo local environment cells to mitochondrial deficient metastatic mammary and melanoma tumor cells in syngenic murine models was shown to restore the respiration of primary and metastatic tumor cells [24]. High relative mtDNA copy number resulting in a high bioenergetic mitochondrial function has been shown to confer an advantage for tumor invasion [25]. Active transfers of mitochondria from human mesenchymal stem cells (MSCs) and skin fibroblasts have also been shown to restore the mitochondrial network of mitochondrial deficient lung adenocarcinoma epithelial cells [26], and mitochondria uptake from MSCs has been shown to promote breast cancer cells OXPHOS and proliferation as well as invasion [27]. Moreover, mitochondrial transfers from bone-marrow MSCs through the endocytic pathway protect leukemia initiating cells potential from ROS-inducing chemotherapy in acute myeloid leukemia [28]. Similar transfers from endothelial cells protect breast and ovarian tumor cells from doxorubicin-induced cell death in vitro [29].

CAFs also reshape mitochondrial network and genome expression in cancer cells. Indeed, primary CAFs have been shown to transfer mitochondria to cancer cells via cellular bridges, also called tunneling nanotubes, in prostate cancer [8] and acute lymphoblastic leukemia (ALL) [30]. Horizontal transfer of functional mitochondria from CAFs enhances prostate cancer cells mitochondrial mass and activity, thus fostering lactate-fueled respiration and further promoting malignancy [8]. In this study, malignant cells that are pre-incubated with CAFs conditioned media are more prone to receive mitochondria, suggesting that a prior education by CAFs is needed in this kind of interaction. Moreover, the transfer of mitochondrial mass in ALL cells from CAFs generated from primary MSCs under ROS-inducing chemotherapy protects cancer cells against the same ROS-inducing agent and is reversed by microtubule inhibition in vivo [30].

Although their frequency and triggering signals need to be further studied, such mitochondrial transfers from CAFs to cancer cells (Figure 1) support the idea of a strong and multifaceted interaction between the two cell types based on mitochondrial processing that promotes tumor malignancy.

### 2.3. CAFs Protect Cancer Cells Mitochondrial Integrity by Regulating Pro- and Anti-Apoptotic Proteins

**Figure 2:**
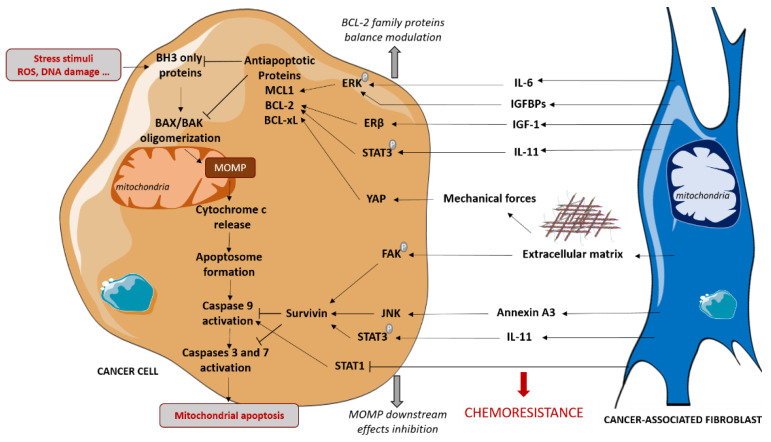
CAFs-mediated protection of cancer cells to mitochondrial apoptosis. CAFs induce chemoresistance in cancer cells by protecting them from mitochondrial apoptosis both by regulating anti-apoptotic proteins level and by limiting caspases activation. These effects are mediated by secretion of cytokines and growth factors and by extracellular matrix production, that is a feature of CAFs. IGF-1: Insulin-like Growth Factor-1, IGFBP: Insulin-like Growth Factor Binding Protein, IL: Interleukin, MOMP: mitochondrial Outer Membrane Permeabilization.

CAFs also protect cancer cells from chemotherapies and specific pro-apoptotic drugs via the modulation of mitochondrial apoptosis-related proteins, favoring tumor escape and proliferation (Figure 2).

Stress stimuli, such as DNA damage or oxidative stress, can trigger BAX and/or BAK oligomerization at the mitochondrial membrane, resulting in mitochondrial outer membrane permeabilization (MOMP). This leads to the cytosolic release of cytochrome c, which induces caspases activation and consequent apoptosis. Stress stimuli alter the equilibrium between pro- and anti-apoptotic BCL-2 family proteins involved in the regulation of BAX and BAK oligomerization. The anti-apoptotic proteins BCL-2, MCL-1 and BCL-xL prevent MOMP via direct interactions with the pro-apoptotic proteins and have been shown to protect cancer cells from stress stimuli induced by chemotherapies [31]. CAFs favor chemoresistance by modulating some BCL-2 family anti-apoptotic protein levels in malignant cells. Indeed, under cisplatin treatment, CAFs promote the phosphorylation and activation of STAT3, which upregulates the levels of BCL-2 in ovarian cancer [32] and in lung adenocarcinoma triggered by IL11 paracrine signaling [33], thus resulting in chemoresistance. CAFs have also been shown to enhance BCL-2 protein levels in bladder cancer cells via the activation of IGF1/ERβ signaling in cancer cells in vitro and in vivo [34]. Moreover, our team showed that CAFs protect luminal breast cancer cells from apoptosis by upregulating the anti-apoptotic MCL-1 in cancer cells via a paracrine IL-6 signaling, which triggers ERK phosphorylation [35]. CAFs-induced MCL-1 upregulation has also been shown to protect breast cancer cells from apoptosis during cell detachment, also known as anoïkis. Indeed, CAFs-secreted IGF-binding proteins trigger the ERK/MAPK pathway in cancer cells and the subsequent inhibition of GSK3 that normally induces MCL-1 degradation [36]. To a larger extent, CAFs have been shown to render HER2+ breast cancer cells less sensitive to apoptosis [37]. In this study, the regulation of apoptotic threshold is implicated in CAFs protective effects to lapatinib, an EGFR/HER2 inhibitor. Notably, the elevation of apoptotic threshold implicates the JAK/STAT signaling pathway in both carcinoma cells and CAFs.

CAFs are a major source of extracellular matrix (ECM) components, such as type I collagen, fibronectin and laminin. MCL1 level has been shown to be upregulated in pancreatic cancer cells cultured on the type I collagen matrix, compared to plastic, conferring resistance to antimetabolite 5-fluorouracil [38]. Moreover, integrins expressed at cancer cells surface trigger survival signals when ligating to ECM components. Indeed, the decrease in BCL-2 protein level induced by paclitaxel treatment is blocked by the integrin-mediated cell attachment of cancer cells to collagen I, fibronectin or laminin, although the induction of signals depends on the cell lines [39]. These results suggest that CAFs could also modulate BCL-2 family proteins in cancer cells via ECM secretion. Interestingly, CAFs conditioned media have been shown to induce the expression of some integrins and BCL-2 in lung carcinoma cells [40]. Notably, integrin B1 and B3 overexpression in malignant cells promotes BCL-2 expression in this study. The mechanical force induced by CAFs-secreted ECM could also be implicated in anti-apoptotic proteins regulation since yes-associated protein (YAP), which can be regulated by mechanical forces [41], has been shown to promote BCL-2 expression in oral squamous carcinoma cells [42].

CAFs also protect cancer cells from chemotherapies by acting downstream of MOMP. Müerköster and colleagues have shown that etoposide resistance of pancreatic cancer cells induced by CAFs in a co-culture model does not rely on pro- nor anti-apoptotic BCL2 family protein regulation [43]. Instead, CAFs epigenetically downregulate caspase expression, inducing transcription factor STAT1, thus limiting caspases 9, 3 and 7 activation. In in vitro and in vivo lung cancer models, CAFs secretion of Annexin A3 has been shown to stimulate cancer cells survivin, known to inhibit caspases activity, thus leading to the decrease in caspases 3 and 8 cleavage under cisplatin treatment [44]. Similarly, CAFs protect lung and ovarian cancer cells from cisplatin via increasing survivin by promoting STAT3 phosphorylation [32,33]. CAFs also protect pancreatic cancer cells from gemcitabine by inducing survivin expression [45]. ECM also acts downstream of MOMP since laminin upregulates survivin by inducing focal adhesion kinase phosphorylation in pancreatic cancer cells, thus promoting chemoresistance [46].

Thus, CAFs have been shown to modulate the expression and activity of anti-apoptotic proteins of the BCL-2 family in cancer cells, resulting in drug resistance. Interestingly, these proteins have been shown to modulate mitochondrial metabolic function in different cell models. Indeed, BCL-2 promotes mitochondrial respiration in cancer cells, resulting in a pro-oxidant state in basal conditions [47], while BCL-xL stabilizes the inner membrane potential and thus modulates mitochondrial energetics in neurons [48]. Moreover, such non-canonical functions of the BCL-2 family anti-apoptotic proteins can depend on their location, where they can interact with metabolic enzymes and transporters. For instance, MCL-1 interaction with outer mitochondrial membrane voltage-dependent anion channel (VDAC) has been shown to increase mitochondrial Ca2+ uptake and reactive oxygen species generation in lung cancer cells [49]. MCL-1 located at the mitochondrial matrix has also been shown to induce respiration in a mouse embryonic fibroblastic model [50]. BCL-xL can also interact with VDAC to favor the open configuration of the channel and metabolite passage through the outer mitochondrial membrane in a murine pro-B lymphocytic cell line [51]. Furthermore, BCL-xL increases adenosine triphosphate ATP production within mitochondria by interacting with the β subunit of F(1)F(0) ATP synthase in neurons [52]. These studies show non-canonical functions of anti-apoptotic BCL-2, BCL-xL and MCL-1, related to mitochondrial function. One can suggest an interconnection between the regulation of mitochondrial function and the regulation of expression or activity of anti-apoptotic proteins of cancer cells in the pro-tumoral effects of CAFs. In particular, it would be interesting to establish whether CAFs modulate BCL-2 anti-apoptotic protein location and interactome with metabolic enzymes and transporters.

## 3. Mitochondrial Processing in CAFs Is Implicated in Their Pro-Tumoral Effects in an Ecosystemic Context

As seen above, CAFs participate in tumor progression via their ability to modulate mitochondrial activity. In this section, we report that this ability relies in part on mitochondrial stress of CAFs triggered by cancer cells, highlighting the reciprocal education between the two cell types. Importantly, mitochondrial processing in fibroblasts participates in their activation. We also discuss the heterogeneity of mitochondrial-mediated interactions between CAFs and cancer cells.

### 3.1. CAFs Mitochondrial Activity Is under Cancer Cells Control

Reciprocal education between CAFs and cancer cells is given in part by their metabolic crosstalk. Glycolytic switch in CAFs which mediates cancer cells metabolic changes is regulated by cancer cells themselves (Figure 1). Indeed, prostate cancer cells induce glycolytic switch in CAFs via the downregulation of mitochondrial deacetylase SIRT3 that promotes oxidative stress and HIF1 stabilization [7]. Oral squamous cell carcinoma cells have also been shown to metabolically reprogram normal oral fibroblasts in an indirect co-culture model by inducing mitochondrial dysfunction reported as ROS accumulation, mitochondrial permeability transition pore opening, hypoxia and mitophagy, associated with an increase in aerobic glycolysis [53]. Moreover, breast cancer cells have been shown to favor CAF oxidative stress via hydrogen peroxide secretion, leading to CAF autophagy and mitophagy mediated by HIF1 stabilization, and promoting mitochondrial dysfunction and enhanced glycolysis [54]. A recent study has shown that triple negative breast cancer cells can induce CAF glycolytic switch and mitophagy via exosome-mediated integrin ITGB4 export that induces ITGB4 expression by CAFs themselves [55]. In these studies, cancer-cell-triggered glycolytic CAFs secrete lactate. Importantly, this secretion is promoted by the upregulation of the monocarboxylate transporter MCT4 in CAFs [7,13,53,55].

Cancer cells, via mitochondrial processing, modulate other CAFs metabolic features involved in their pro-tumoral effects. Under stiff matrix conditions, squamous cell carcinoma cells have been shown to secrete glutamate that can be used by CAFs to fuel the TCA cycle and produce aspartate, that, in turn, fuels cancer cells for nucleotides biosynthesis, favoring tumor growth [56]. Notably, cancer-cell-secreted glutamate induces the glutathione pathway in CAFs, thus limiting the accumulation of ROS and superoxide induced by matrix stiffness. In a glutamine deprived co-culture ovarian cancer model, malignant cells have been shown to enhance TCA cycle activity in CAFs to maintain glutamate and citrate levels for glutamine synthesis, that is then secreted by CAFs to support cancer cells proliferation [11].

Altogether, CAFs metabolism that is involved in cancer cells mitochondrial processing is controlled by cancer cells themselves within a reciprocal mitochondrial education.

### 3.2. Mitochondrial Dynamics Is Involved in Fibroblasts Activation

Although CAFs can arise from bone marrow-derived precursors, mesenchymal stem cells, or endothelial cells, resident fibroblasts have been described as the major source of CAFs [57].

Mitochondrial activity and dynamics have been particularly implicated in TGF-β signaling leading to fibroblast activation into myofibroblasts, characterized by αSMA expression and invasive and migratory abilities. In normal human lung fibroblasts, mitochondrial ROS generated at mitochondrial respiratory chain complex III are required for TGF-β-induced gene expression, in particular, αSMA [58]. High mitochondrial generation of ROS caused by respiratory chain complex I dysfunction also correlates with myofibroblast activation [59]. TGF-β is not the only activation signaling modulated by mitochondria, since mitochondrial ROS have also been shown to regulate PDGF-induced signaling in primary mouse embryonic fibroblasts via oxidation of protein tyrosine phosphatases [60], possibly promoting fibroblast activation. Moreover, mitochondrial dynamics are involved in TGF-β signaling, since targeting mitochondrial-fission-mediator DRP-1 inhibits TGF-β-induced rat kidney fibroblast cell activation [61].

Interestingly, the acquisition of CAFs’ metabolic characteristics by normal fibroblasts co-cultured with malignant cells precedes the acquisition of the fibroblast activation protein (FAP) and loss of Caveolin 1 [53]. This result suggests that metabolic reprogramming could participate in the activation of fibroblasts by cancer. Concordant with this, TGF-β-induced early increase in glycolysis in lung fibroblasts sustains transformation into myofibroblasts. More precisely, glycolysis increases the TCA cycle intermediate succinate, which stabilizes HIF1α and promotes myofibroblastic differentiation [62], thus implicating metabolic mitochondrial function in TGF-β-induced fibroblast differentiation. Reduced mitochondrial α-ketoglutarate has also been shown to stabilize HIF1α under normoxia in human colon CAFs under TGF-β or PDGF stimulation, thus favoring glycolysis. Of note, albeit no consensus was found, HIF1α has been shown in several studies to permit oncogenic gain of functions in fibroblasts [63]. These results highlight the essential role of mitochondria in fibroblast activation.

Thus, fibroblast mitochondrial activity participates in the signaling pathways, leading to their activation. Within tumors, the fibroblast metabolic switch could either be an active phenomenon favoring their activation or a consequence of their activated state.

### 3.3. Mitochondrial-Mediated Interactions between CAFs and Cancer Cells Are Heterogeneous

CAFs exert specific metabolic and mitochondrial processing in cancer cells depending on cancer types and subtypes. As described earlier, the nature of the identified secreted nutrients and subsequent metabolism seems to vary according to the localization of the cancer—lactate in prostate cancer, lactate/pyruvate in breast cancer, alanine in pancreatic cancer and glutamine in ovarian cancer. This heterogeneity could originate from the specific metabolism of cancer cells [64], that could be a factor of strong interaction between cancer cells and CAFs. Additional to these disparities between cancer, depending on the tissue they originate from, intra-tumoral heterogeneity could be a source of varying cancer cells/CAFs interactions. As previously reviewed by Strickaert and colleagues [65], the concept of tumor heterogeneity includes the diversity of the cell populations, including stromal cells, the cell location within the tumor, the epigenetic and genetic effects in cancer cells over time and, eventually, the variation in metabolism, all of these being strikingly linked to each other. Concerning cell location within the tumor, by using a micro-patterned co-culture model consisting in a breast cancer cell (MCF7) island surrounded by stromal cells, it has been shown that stromal mechanical constraints induce spatial heterogeneity of mitochondrial activities in cancer cells, with an impact on metabolism and the metastatic potential of cancer cells [66]. On the other hand, in melanoma, it has been reported that heterogeneity in MCT1-high and MCT1-negative or low expressing cancer cells discriminate their metastatic potential. MCT1-high cells uptake more lactate and are more efficient to metastasize, implying that glycolytic CAFs exert a differential pro-metastatic effect on these melanoma cell sub-populations [67]. Moreover, metabolic heterogeneity has been shown within malignant cells of mammary tumors by single-cell transcriptomics in a MMTV-PyMT mouse model [68]. Indeed, one PyMT cell subpopulation expresses higher levels of genes involved in OXPHOS, while another shows higher glycolytic process gene expression. Intra-tumoral malignant cell metabolic heterogeneity could either be the result of the interactions with CAFs or could directly modulate this interaction, resulting in different pro-tumoral effects. In human breast cancer, metabolic interaction between cancer and stromal cells could further vary according to molecular subtype [69]. Indeed, this immunohistochemical study suggests a correlation between the subtype and the metabolic phenotype of the tumor (Warburg type with glycolytic tumor cells and non-glycolytic stroma or Reverse Warburg type with non-glycolytic tumor cells and glycolytic stroma). Moreover, the secreted intermediates that modulate mitochondrial function are specific of certain subtypes of cancer. It seems the case with breast-cancer-cell-secreted ITGB4 that it is mainly secreted by triple negative breast cancer cell lines, and more specifically by some of the lines of this molecular subtype [55].

The heterogeneity of the mitochondrial-mediated dialogue between CAFs and cancer cells can also be highlighted by the heterogeneity of mitochondrial activity of CAFs within tumor. CAFs are indeed heterogeneous within tumors, which could be explained by their adaptability to their environment. Determining whether CAFs mitochondrial activity also depends on their diverse cellular origins [70] would be of particular interest. Costa and colleagues identified four subsets of CAFs within breast tumors from patients [71]. RNA sequencing shows that one subset, called S4 and characterized among others by high αSMA expression and low FAP expression, exhibits gene enrichment in oxidative metabolism. The four subsets have also been identified in ovarian cancer [72]. In this latter model, the S4 subset exhibits a strong enrichment in genes encoding electron transport chain proteins. Moreover, Qian and colleagues recently identified metabolic heterogeneity between CAFs subpopulations commonly found in colorectal, ovarian and lung tumors, with some populations characterized by glycolytic signature, based on single-cell analysis of transcription factor activities [73]. These studies suggest heterogeneity in CAFs mitochondrial function within the tumor. Costa and colleagues also show specific spatial distribution with subsets S1 and S4 preferentially accumulating in the tumor while the other subtypes are found in juxta-tumors. Of note, CAFs present in juxta-tumors are enriched in CAFs with genes involved in oxidative stress, potentially revealing mitochondrial dysfunction. These results suggest that spatial proximity to the tumor could be important for CAF mitochondrial function. CAFs have indeed been shown to adapt their metabolism to the nutritional context [74], that is mainly influenced by cancer cells metabolic activity.

Thus, the interactions between CAFs and cancer cells and the consequences on mitochondrial functions depend on cancer type and seem heterogeneous within tumors, emphasizing the complexity of the understanding of the dialogue between the two cell types.

## 4. Targeting Mitochondria to Counteract CAF-Cancer Cells Symbiosis in Tumor Ecosystems

Targeting the tumor-stroma symbiotic crosstalk is an emerging strategy in cancer therapy. As a core platform in the pro-tumoral dialogue between CAFs and cancer cells, mitochondria must be a key target to improve current therapies in cancers. Among the targetable mitochondrial function in tumor ecosystems, we focus here on the two major pathways presented in the previous sections, namely energy metabolic activity and apoptosis. Besides their impact on tumor progression, mitochondrial generation of energy and valuable intermediates, glycolysis and oxidative phosphorylation promote therapy resistance [75]. By their ability to prevent mitochondrial apoptosis, one of the causes of the limited therapy efficacy and drug resistance, BCL-2 family members arise as potential targets in cancers.

### 4.1. Targeting Metabolites Bidirectional Exchanges between CAFs and Cancer Cells

As largely described, glycolytic CAFs can sustain cancer cells with lactate (and its derivative—e.g., pyruvate) to fuel mitochondrial activity and promote cancer progression [4,7,8,9]. One promising drug to limit lactate shuttle between glycolytic CAFs and cancer cells is dichloroacetate (DCA) [76]. DCA, by inhibiting pyruvate dehydrogenase kinase (PDK) enhances the pyruvate dehydrogenase (PDH) activity and increases the subsequent entry of pyruvate into the Krebs cycle. It results in a decrease in lactate secretion and aerobic glycolysis. It was recently shown that extinction of PDK in αSMA+ fibroblasts co-injected with 4T1 cells in an orthotopic tumors model decreases tumor growth [77]. In the same study, the authors show that breast cancer derived human CAFs are pro-glycolytic compared to fibroblasts from benign lesions. This reinforces the idea that therapeutic agents targeting glycolysis, such as DCA or 2-Deoxy-D-glucose (2DG), could be used to counteract metabolic fueling of cancer cells by CAFs. Thus, combined treatments with DCA and 2-DG block the tumor growth of MDA-MB231 induced by the highly glycolytic Caveolin-/- fibroblasts [78]. Nevertheless, the anti-tumoral effect of DCA on cancer cells is controversial due to its various impact on different models, such as in colonic cancers [79]. Further studies need to be conducted to precise the different pyruvate metabolic pathways in cancer cells under CAFs influence. This should lead to identify the metabolic phenotype in which forcing cells to perform OXPHOS with DCA is a benefit to alleviate the tumor growth.

The reciprocal transfer of lactate from cancer cells to CAFs has also been shown to enhance tumor progression and therapy resistance, making the monocarboxylate transporters (MCTs) responsible for lactate exchange, a promising target in cancers. Lactate and lactate derived metabolites cell–cell shuttle occurs via MCT1-MCT4 against which different inhibitors have been developed, such as α-cyano-4-OH-cinnamate (CHC) (Pan inhibitor), AR-C155858 (MCT-1/2 inhibitor), and AZD3965 (MCT-1 inhibitor) [80]. Although AZD3965 or MCT-1/4 silencing drastically decreases tumor growth in xenograft tumor models, whether or not their efficacy results in a disruption in CAF-cancer cells dialogue has not been established [81,82]. Nevertheless, Fiaschi and colleagues show, in vitro and in vivo, that CAFs’ supporting role on prostate cancer cell growth is drastically reversed by CHC treatment or MCT-1 silencing in cancer cells [7]. MCT-1 also enables pyruvate transfer, which has been shown to be secreted by CAFs to fuel breast cancer cells [83]. Therefore, in glycolytic breast cancer cells, MCT-1 inhibition with AZD3965 results in disruption of pyruvate transport rather than lactate and is accompanied by an increase in intracellular pyruvate concentration and oxygen consumption rate [81]. These results raise the question of targeting pyruvate metabolism and especially mitochondrial pyruvate transport. In this way, Feron’s team identified 7ACC2, a compound initially described to specifically block lactate influx, as a mitochondrial pyruvate transport inhibitor. This study shows that 7ACC2 slows down tumor growth by sequentially disrupting mitochondrial pyruvate entry, generating intracellular pyruvate accumulation and limiting lactate influx [84]. This suggests that blocking the mitochondrial pyruvate carrier could have a strong cytotoxic effect by simultaneously inhibiting lactate uptake and mitochondrial respiratory metabolism. Interestingly, the blockade of lactate/pyruvate exchange by increasing glycolysis and oxygen availability improves sensitivity to radiotherapy [84,85].

Numerous studies show an increase in oxidative phosphorylation in cancer cells under pressure from microenvironmental CAFs. As reported above in Section 2.1, CAF-induced OXPHOS activity in cancer cells is related to amino-acid supply, such as glutamine in ovarian tumor or alanine in pancreatic ductal adenocarcinoma (PDAC), of the latter by the former [10,11]. Unfortunately, monotherapy using glutaminase inhibitor fails to impact tumor growth in vivo [86]. Yang and colleagues showed that blocking glutamine synthesis in CAFs and glutamine catabolism in cancer cells improves therapeutic outcomes in an orthotopic mouse model of ovarian carcinoma [11]. The blockade of amino acid exchange in metabolic support from CAFs to cancer cells remains a therapeutic challenge as Parker and colleagues identify SLC38A2 as a unique critical alanine transporter in PDAC tumorigenesis [87].

In addition to targeting specific shuttles to counteract metabolic symbiosis between CAF and cancer cells, alternatively targeting the resulting metabolic reprogramming in cancer cells could be completed. Direct OXPHOS targeting in tumor–stroma metabolic dialogue could also be achieved using metformin, which functions in part through the inhibition of mitochondrial respiratory chain activity or the activation of AMP activated protein kinase [88]. By inducing cancer cell death and improving sensitivity to other therapies, the potential use of metformin in tumor treatment is attractive. Metformin disrupts tumor-stromal crosstalk by inactivating CAF and preventing their subsequent secretion of pro-tumoral factors such as SDF-1 and IL-8 in breast cancer or IL-6 in ovarian cancer [89,90]. The two latter studies show only an effect on tumor growth when CAF have been pretreated but the effect of metformin on cancer cells in their micro-environment was not investigated. Indeed, the efficacy of metformin in cancer treatment is not conclusive. One explanation could be that, although metformin reverses the CAF-activated state without inducing cell death, normal fibroblasts could by themselves block metformin induced cancer cell apoptosis [91]. The lack of efficacy of metformin as a single agent has also been retrieved in a model of pancreatic microtumors, consisting in MiaPaCa2 cells and patient derived CAFs in 3D spheroids, despite a significant reduction in redox states. However, adjuvant treatment with metformin overcomes resistance to oxaliplatin or photodynamic therapy induced by CAFs [82].

### 4.2. Targeting Mitochondrial Apoptosis

Most anticancer drugs currently used in clinical oncology exploit mitochondrial apoptotic signaling pathway to trigger cancer cell death. Cancer cells can escape apoptosis by modifying the equilibrium between the anti- and pro-apoptotic members, including BH3-only proteins of the BCL2 family. Small molecules, named BH-3 mimetics, which functionally replicate the pro-apoptotic effect of BH3-only proteins and can therefore specifically counterbalance the effect of pro-survival proteins (BCL-2, BCL-xL and MCL-1), have been developed. We reported above in Section 2 that CAFs can help malignant cells to escape cell death by modulating sensitivity to apoptosis in these cells [35,37]. Thus, BH-3 mimetics provide opportunity to counteract CAF induced chemoresistance. Consistently, using synthetic lethality screen, Marusyk and colleagues identify BCL-2/BCL-xL inhibitors (ABT-737 and ABT-263) as candidates to overcome CAF-induced resistance to lapatinib in breast cancers [37]. In luminal breast cancer cells, we have shown that CAFs conditioned-media-induced apoptotic resistance to BCL-2/BCL-xL inhibitors could be completely reversed with a BH-3 mimetic targeting MCL-1 (A1210477). Of note, the combination of the two inhibitors ABT-737 and A-1210477 leads to the death of both cancer cells and CAFs [35].

Regarding the pro-tumorigenic effects of CAFs, it is tempting to directly target CAFs in therapy by inducing apoptosis, provided the strategy selectively kills activated fibroblasts. The ability of BH-3 mimetics to trigger apoptosis is governed by the “primed for death” state of the cells [92]. Nevertheless, due to CAFs heterogeneity, prior to be therapeutically exploited, a precise knowledge of the apoptosis priming of the distinct CAFs subsets, including pro-tumoral and anti-tumoral populations, is needed. As it is, there are differences in mitochondrial priming between normal and activated fibroblasts, but also between CAFs, depending the type of cancers they originate from. Using the BH3 profiling technique, it has been suggested in a fibrosis model that activated myofibroblast is primed to apoptosis, as targeting BCL-2/BCL-xL with ABT-263 induces apoptosis in fibroblasts exclusively when they are mechano-activated. The forced expression of αSMA in fibroblasts led to the same results by inducing BCL-xL expression in a YAP-TAZ dependent manner [93]. We also showed that contrary to normal fibroblasts, breast CAFs rely on MCL-1 for their survival. This could be related to their activated state as normal human lung fibroblasts become sensitive to MCL-1 inhibition following their “activation” by TGF-β concomitantly with αSMA expression and MCL-1 protein stabilization [35]. Differently, in cholangiocarcinoma, cancer-cell-secreted PDGF (previously reported to promote CAF activation) was shown to induce apoptotic priming of CAFs. It is likely to occur by the facilitation of binding of Bak to BCL-2, which renders the CAFs more sensitive to ABT-263 [94]. In accordance with this, ABT-263 treatment depletes CAFs, reduces tumor burden and decreases lymphatic vascularization and metastasis in cholangiocarcinoma in vivo [94,95,96]. Although dependence of apoptosis priming to the CAFs activated state seems fairly consensual, it remains that, depending on the tissue, CAFs rely on different anti-apoptotic proteins for their survival. Whether BH-3 mimetics modulate stroma aggressiveness by targeting distinct CAF subpopulations within the tumor remains an open question.

## 5. Conclusions

Emerging therapeutic strategies aim to overcome CAF-cancer cells’ pro-tumoral symbiosis on the one hand and to target mitochondria in cancers on the other hand. Numerous studies address the pro-tumoral metabolic reprogramming of cancer cells by CAFs and identify attractive pharmacological targets. Nevertheless, the preclinical validation of these targets in a CAF-cancer cell context remains poorly investigated. Moreover, metabolic drugs are controversial because of their off-target effects on normal cells. Due to the obvious reciprocal influence between CAFs and cancer cells regarding their mitochondrial functions—i.e., metabolic activity and apoptosis resistance—it might be more efficient to target the two cellular processes in a context of tumoral ecosystem. However, both therapeutic options are limited by the heterogeneity of CAFs/cancer cell dialogue and apoptotic priming, originating from cancer subtype and stage, and presence within the tumor. This emphasizes the importance of models used to study mitochondrial implications in tumor–stroma mutual support. Finally, the understanding of close interactions between cellular metabolic features and mitochondrial apoptosis could help to find better targets in cancer therapies.

## Abbreviations

ATP	Adenosine TriPhosphate
CAF	Cancer-Associated Fibroblast
ECM	ExtraCellular Matrix
EMT	Epithelial-Mesenchymal Transition
ETC	Electron Transport Chain
GSH	reduced glutathione
HIF	Hypoxia Inducible Factor
IGF	Insulin-like Growth Factor
ITGB4	Integrin Beta 4
MCT	MonoCarboxylate Transporter
MOMP	Mitochondrial Outer Membrane Permeabilization
mtDNA	mitochondrial DNA
PGC1α	Peroxisome proliferator-activated receptor gamma coactivator 1-alpha
ROS	Reactive Oxygen Species
TAZ	Transcriptional coActivator with PDZ-binding motif
TCA	TriCarboxylic Acid
YAP	Yes-Associated Protein
